# Computational Characterization of Transient Strain-Transcending Immunity against Influenza A

**DOI:** 10.1371/journal.pone.0125047

**Published:** 2015-05-01

**Authors:** David C. Farrow, Donald S. Burke, Roni Rosenfeld

**Affiliations:** 1 Lane Center for Computational Biology, Carnegie Mellon University, Pittsburgh, PA, 15213, United States of America; 2 Graduate School of Public Health, University of Pittsburgh, Pittsburgh, PA, 15260, United States of America; 3 Joint Carnegie Mellon University—University of Pittsburgh Ph.D. Program in Computational Biology, Pittsburgh, PA, United States of America; Harvard School of Public Health, UNITED STATES

## Abstract

The enigmatic observation that the rapidly evolving influenza A (H3N2) virus exhibits, at any given time, a limited standing genetic diversity has been an impetus for much research. One of the first generative computational models to successfully recapitulate this pattern of consistently constrained diversity posits the existence of a strong and short-lived strain-transcending immunity. Building on that model, we explored a much broader set of scenarios (parameterizations) of a transient strain-transcending immunity, ran long-term simulations of each such scenario, and assessed its plausibility with respect to a set of known or estimated influenza empirical measures. We evaluated simulated outcomes using a variety of measures, both epidemiological (annual attack rate, epidemic duration, reproductive number, and peak weekly incidence), and evolutionary (pairwise antigenic diversity, fixation rate, most recent common ancestor, and kappa, which quantifies the potential for antigenic evolution). Taking cumulative support from all these measures, we show which parameterizations of strain-transcending immunity are plausible with respect to the set of empirically derived target values. We conclude that strain-transcending immunity which is milder and longer lasting than previously suggested is more congruent with the observed short- and long-term behavior of influenza.

## Introduction

Despite much research and efforts toward prevention in recent times, influenza remains a significant cause of disease burden worldwide [1]. A primary obstacle in the search for a long-term vaccine is the distinctive phylodynamics of the influenza viruses, driven by short infection periods and intermediate levels of cross-immunity in humans [2]. Influenza A/H3N2 in particular is responsible for the majority of new cases of seasonal influenza [3] and exhibits a high fixation rate in comparison to other types and subtypes [4,5]. With a rapid rate of evolution and a continual selective pressure to evade host immunity, influenza could be reasonably expected to exhibit sustained and saturating genetic diversity, as has been observed in the case of other RNA viruses including HIV and HCV [2]. Yet the opposite appears to be the case; since its pandemic appearance in 1968, A/H3N2 has maintained low genetic diversity and has given rise to a generally linear phylogeny, consisting of a single, well-defined trunk and short-lived side branches (see Figure 1 of [6]). This enigmatic observation has been an impetus for much modeling and research in recent years.

The modeling of influenza's long-term evolution has been approached from many directions with a wide variety of different techniques. Both mathematical and individual-based models have seen a large degree of success in recapitulating the distinctive phylodynamics of the virus. One of the earliest and most prominent of these models hypothesizes the existence of a short-lived, strain-transcending immunity in humans to explain the limited diversity of influenza [4]. A prominent alternative hypothesis instead suggests that epochal evolution is sufficient to constrain diversity; this was originally modeled using a neutral network genotype-to-phenotype map [7] and was later generalized [8]. Other hypotheses of influenza evolution posit an extremely limited set of antigenic phenotypes [9] or suggest that antigenic evolution is canalized by human immunity [10]. Though all of these ideas have contributed greatly to the debate over the mechanisms driving influenza's phylodynamics, it is transient strain-transcending immunity in particular that we study herein.

Transient strain-transcending immunity is defined in [4] as protection against all strains, decaying exponentially over time, acquired upon infection with any individual strain. It is specified by two parameters: the initial *strength* of protection (from completely ineffective to completely protective), and the *half-life* of its decay (any duration of time). Its classical, pragmatic parameterization—completely protective and ephemeral (on the order of months)—has seen widespread adoption within models of influenza [6,11,12,13], and it has been argued that the degenerate case of weak, lifelong strain-transcending immunity manifests as a uniform reduction in host susceptibility and fails to function as a density-dependent constraint on diversity [4]. Existing somewhere between these two extremes of efficacy and temporality, the intermediate regimes of strain-transcending immunity have hitherto remained largely unconsidered. While there is limited biological evidence in support of strain-transcending immunity [14], experimental validation of its presence in humans or animals remains a major challenge, significantly exacerbated by lack of prior constraints on its efficacy and the timescales of its duration. Therefore, we computationally explore all practical regimes of transient strain-transcending immunity to determine which are plausibly consistent with influenza, using an approach similar to Approximate Bayesian Computation (ABC, [15]) to evaluate the likelihood of each parameterization.

## Results

The parameter space of transient strain-transcending immunity as defined in [4] is a two-dimensional surface with *strength* spanning from 0 to 1 (completely ineffective to completely protective, respectively) and *half-life* spanning from instantaneous to the order of host lifespans (effectively permanent). For practical considerations and reasons discussed above, we constrained the exploration to the region bounded by strength from 0.25 to 1.00 and half-life from 41 days to 60 years, reasoning that exceedingly weak or short parameterizations approach the degenerate case of nonexistence of strain-transcending immunity (see S1 Supporting Information). Over this bounded space we imposed a grid of 60 points (roughly equally spaced in strength and in the logarithm of half-life) to represent a large set of potential parameter regimes of strain-transcending immunity.

To sample a given point in this parameter space requires a realization of the model. To this end we implemented the model as described in [4] with only slight deviations in parameterization (namely, larger population size and longer host lifespan) to take advantage of recent increases in computing power (see Methods). Using this simulator, we sampled each grid point 20 times, each sample being a 50 year simulation of influenza evolution in a population of 100 million human hosts. We then measured from simulation output the following properties at each grid point. (Below, ‘±’ indicates a 95% credible range.)

**Fig 1:**
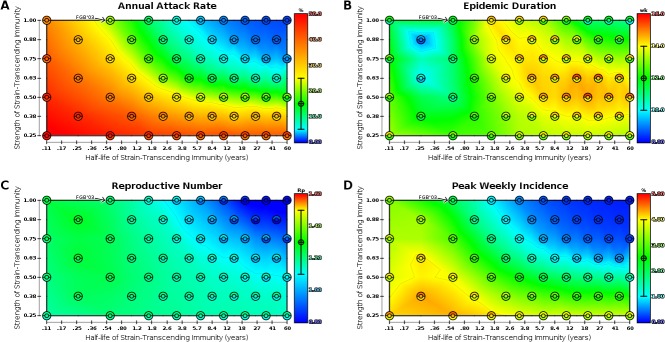
Epidemiological parsimony is measured across transient strain-transcending immunity parameter space. Sixty parameterizations of strain-transcending immunity (strength and duration) were simulated. Within each map, the 60 parameterizations are represented by a set of circles and semicircles; the inner circle at each point represents the sample mean of the measure, and the top and bottom semi-circles represent the mean plus and minus one sample standard deviation, respectively (n = 20 realizations for each point). Color corresponds to the agreement between the simulated outcome and the expected outcome for influenza; blue indicates a value below the 95% CI for influenza, red indicates a value above the 95% CI for influenza, and green represents the expected value for influenza. The 95% CI for influenza is marked on each color scale, and the target value is indicated by ‘⊕’. The tip of the “FGB’03” arrow indicates the default parameterization given in [4]. Triangulation and interpolation were used to achieve smooth shading throughout the space to facilitate visual identification of spatial trends. Faint contour lines demarcate confidence interval boundaries. **(A)** AAR measured from model output; target value is 0.15 (95% CI: ±0.10). **(B)** Epidemic duration measured from model output; target value is 12 (95% CI: ±2) weeks. **(C)** R_p_ measured from model output; target value is 1.3 (95% CI: ±0.2). **(D)** Peak weekly incidence measured from model output; target value is 0.025 (95% CI: ±0.015).

**Annual Attack Rate** (AAR) is the number of individuals infected during a single year, averaged over all years. This is effectively the cumulative incidence over a period of one year and is typically measured as the percentage of the population that becomes infected during the epidemic. Several estimates of the AAR of influenza are available in the literature [3,16,17,18,19,20] and give typical values ranging from 5% to 25%, depending on age; we set our age-independent target value to 15% (±10%) on these estimates. In our model, AAR is closest to the target value when strength and duration of strain-transcending immunity are both relatively high, with some degree of tradeoff (Fig 1A).

**Epidemic Duration** is a measure of how long seasonal epidemics last, averaged over all seasons. We defined this as the range of weeks, containing the week of peak incidence, that captures 90% of the seasonal attack rate. Based on our estimate of the empirical epidemic duration for the period spanning July 1, 2003 to July 1, 2012 using U.S. surveillance data available on FluNet [21], and in accordance with similar estimates available in the literature [16,17,20,22,23], we set a target average epidemic duration of 12 (±2) weeks. In our model, average epidemic duration is a complex function of strain-transcending immunity, but in general is close to the target value for moderate durations of strain-transcending immunity (Fig 1B).

**Reproductive Number** (R_p_) is a dimensionless number that quantifies the expected number of secondary cases arising from a primary case throughout the duration of an infectious period. This quantity differs from the basic reproductive number (R_0_) in that R_0_ assumes a completely naive population, whereas R_p_ assumes a population with some pre-existing partial immunity [18]. In the case of influenza, where a significant portion of the population has lingering immunity from strains encountered either during a previous season or through vaccination, R_p_ is a more appropriate measure than R_0_. R_p_ for seasonal influenza in temperate regions has been estimated to be 1.3 (95% CI 1.2–1.4) [18,24], though here we assumed the more permissive range of 1.1–1.5 to be credible. In our model, R_p_ is closest to the target value when strain-transcending immunity is strong and short-lived (Fig 1C).

**Peak Weekly Incidence** is the peak of the weekly incidence of each seasonal epidemic, averaged over all seasons. The true peak incidence of influenza is difficult to measure, so instead we base our target range on estimates from the previously mentioned individual based models. Specifically, we estimate based on the model described in [4] the peak weekly incidence under normal epidemic conditions to be 2,500 (±1,500) hosts per 100,000 hosts, or 2.5% (±1.5%). In our model, peak weekly incidence is closest to the target value when strength and duration of strain-transcending immunity are inversely proportional (Fig 1D).

Up to this point we have focused solely on the epidemiological values derived from weekly incidence counts, measuring height (peak weekly incidence), area under the curve (AAR), width (epidemic duration), and shape (R_p_). Now we shift our attention to the group of measures that quantify the evolutionary trajectory of the virus.

**Fig 2:**
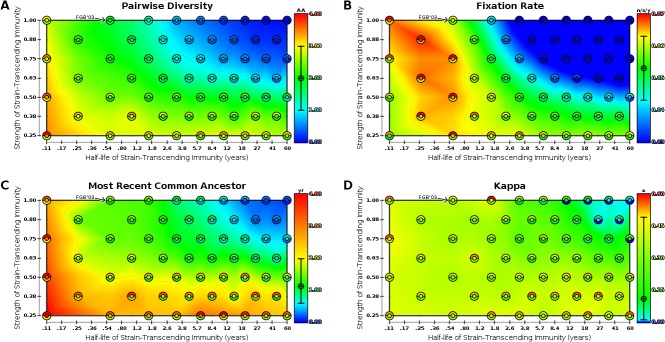
Evolutionary parsimony is measured across transient strain-transcending immunity parameter space. Maps are as described in Fig 1. **(A)** Antigenic diversity measured from model output; target value is 2 (95% CI: ±1) amino acids. **(B)** Fixation rate measured from model output; target value is 0.053 (95% CI: ±0.01) substitutions per site per year. **(C)** MRCA measured from model output; target value is 1.12 (95% CI: 0.58–1.97) years. **(D)** κ measured from model output; target value is 0.11 (95% CI: 0.01–0.49).

**Pairwise Diversity** is the prevalence weighted mean pairwise number of amino acid differences between all pairs of strains existing on some day, averaged over all days. It has been previously used to quantify the average amount of viral antigenic [4] and genetic [7] diversity and is an indicator of whether viral diversity is constrained (consistent with influenza) or unconstrained (inconsistent with influenza). Because it is the average number of amino acid differences between two strains, the possible values range from 0 to the number of amino acids modeled, 12 in these simulations. Though the diversity of influenza follows a ‘boom-and-bust’ pattern [7], we expect that pairwise diversity should, on average, remain low. Therefore, we assumed the plausible target value for these modeled strains to be 2 (±1) amino acids. In our model, pairwise diversity is closest to the target value when strength and duration of strain-transcending immunity are inversely proportional (Fig 2A).

**Fixation Rate** is a measure of how quickly the virus evolves, as indicated by the number of novel mutations becoming fixed in the viral phylogeny over some period of time. The mutation rate for the A/H3N2 subtype is particularly high [5] and, for the sites assumed to be under positive selection, has been measured to be 0.053 (±0.01) substitutions per site per year [4]. In our model, fixation rate is closest to the target value when strength and duration of strain-transcending immunity are inversely proportional (Fig 2B).

**Most Recent Common Ancestor** (MRCA) measures the number of years separating all contemporaneous strains. Here we used the average number of years of evolution separating two randomly sampled strains on any given day to approximate the MRCA, as in [6,10]. The target value of for influenza, using the HA gene only, is roughly 1.12 years with a 95% confidence interval roughly spanning 0.58 to 1.97 years (excluding the 2002–03 season outlier) [22]. In our model, MRCA is closest to the target value when strength of strain-transcending immunity is moderate or high; this measure appears to be somewhat insensitive to changes in duration of strain-transcending immunity (Fig 2C).

**Kappa** (κ) is a dimensionless number which quantifies the potential for antigenic evolution of rapidly evolving viruses [25]. We use Kappa to probabilistically quantify where on the diversification spectrum, from very constrained (as expected for A/H3N2 flu) to exceedingly diverse (as observed for between-host HIV), a simulated phylogeny falls. Kappa is measured here as the parameter of the best fit Poisson distribution (determined by maximum likelihood estimation) given the counts of excess antigenic variants, per variant, over the duration of the simulation. With its characteristic phylogeny, influenza exhibits a relatively low degree of phylogenetic branching, and κ has been estimated to be 0.11 with a 95% confidence interval roughly spanning from 0.01 to 0.5 based on [25]. In our model, κ is closest to the target value when strain-transcending immunity is relatively strong over a moderate duration (Fig 2D).

**Fig 3:**
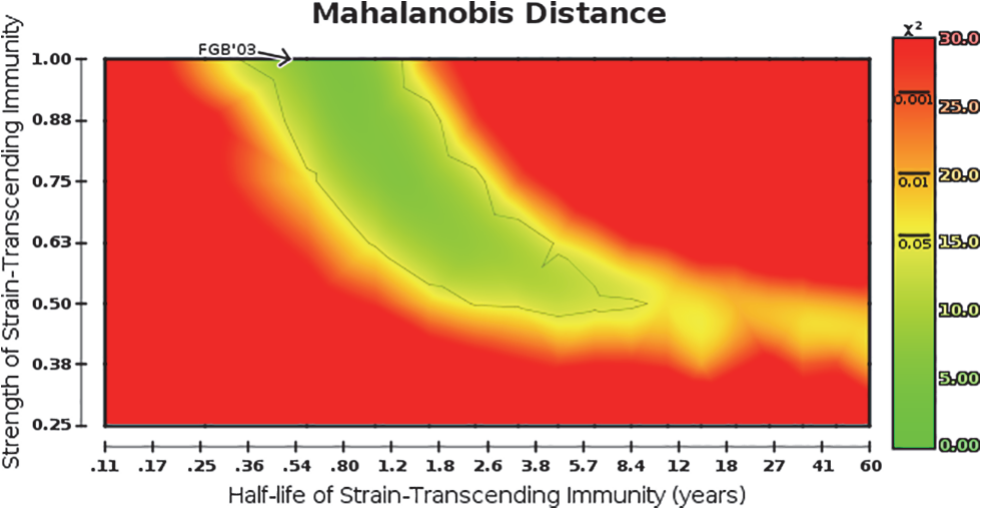
Transient strain-transcending immunity is potentially longer lasting and weaker than previously suspected. Map is as described in Fig 1 with circles at each individual parameterization suppressed. Color encodes the value of the chi-squared test statistic; green represents inability to reject the null hypothesis that an outcome is *not* a multivariate outlier (equivalently, *is* influenza-like), and red represents high confidence in the rejection of that hypothesis. Lines on the color scale indicate probability cutoffs at the p≤0.05, p≤0.01, and p≤0.001 levels for a chi-squared distribution with eight degrees of freedom.

Having examined each measure in isolation (summarized in Table 1; see S1 Supporting Information for algorithms), we then aimed to characterize the plausible range of parameterizations of strain-transcending immunity based on the cumulative results of all eight measures. Plausibility was determined by testing, for each grid point separately, the null hypothesis that a combined set of measured outcomes are consistent with empirically-derived targets for influenza. Herein, we made the simplifying assumptions that all measures are independent and empirical targets are normally distributed. We performed additional analysis to determine how these assumptions affect results and found that the shape and location of the plausible region in parameter space are essentially unaffected by these assumptions (see S1 Supporting Information). At each point in parameter space, a Mahalanobis distance [26] was calculated and compared to a chi-squared distribution with 8 degrees of freedom. The grid points which significantly deviated from expected outcomes were labeled as implausible. The remaining grid points constitute the plausible region, extending from a few months to a decade in duration and from 100% to 50% strength (Fig 3), and suggest that strain-transcending immunity in humans could potentially be much longer lasting and far weaker than previously assumed.

**Table 1:** All epidemiological and evolutionary measures summarized.

Measure	Target Value	95% CI	Source
Annual Attack Rate (AAR)	15%	5–25%	[3,16,17,18,19,20]
Epidemic Duration	12 weeks	10–14 weeks	[16,17,20,22,23]
Reproductive Number (R_p_)	1.3	1.1–1.5	[18,24]
Peak Weekly Incidence	2.5%	1–4%	Following [4]
Pairwise Diversity	2 AA	1–3 AA	Following [4]; see also [7]
Fixation Rate	0.053*	0.043–0.063*	[4]
Most Recent Common Ancestor (MRCA)	1.12 years	0.58–1.97 years	[22]; see also [6,10]
Kappa (κ)	0.11	0.01–0.5	Based on [25]

* Number of nucleotide substitutions per site per year.

## Discussion

**Fig 4:**
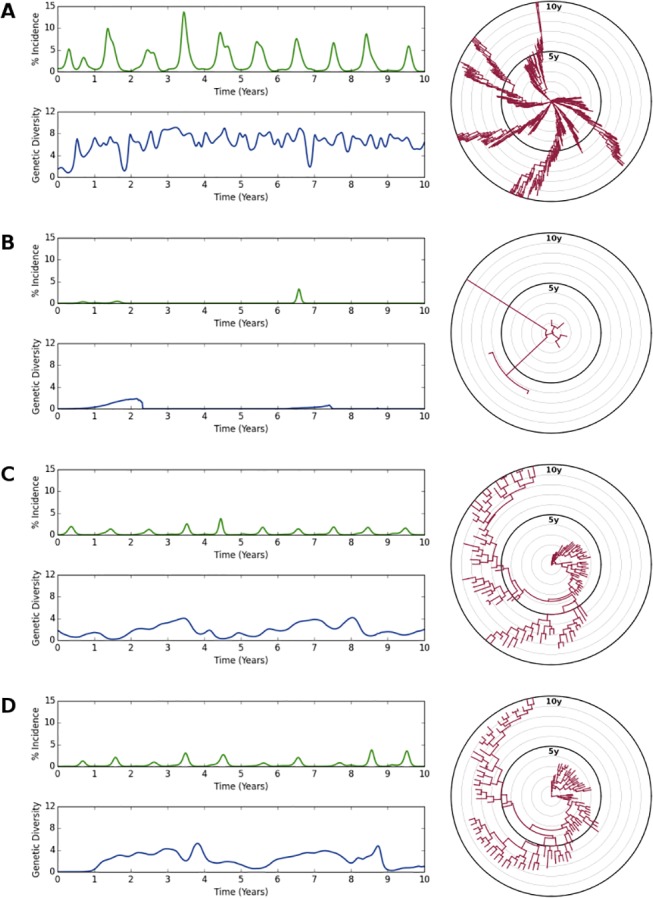
Characteristic epidemiological and evolutionary dynamics are observed within different parameter regimes. For each parameterization, a 10 year excerpt from a typical simulation is shown. Graphs on the left show weekly percent incidence (northern hemisphere only, green) and weekly mean pairwise antigenic diversity (blue). Radial dendrograms on the right show phylogeny (red) with concentric rings marking one year intervals. **(A)** Weak and short-lived strain-transcending immunity (strength = 25%, half-life≈1.3 months). Peak incidence and attack rate are greatly elevated; diversity is unconstrained; and phylogeny is exceedingly branched. **(B)** Strong and long-lived strain-transcending immunity (strength = 100%, half-life≈11 years). Sporadic outbreaks result in greatly reduced peak incidence and attack rate; antigenic diversity approaches zero during extended periods of near-extinction; and phylogeny is exceedingly slender. **(C)** Parameterization similar to that described in [4]: strong and short-lived strain-transcending immunity (strength = 100%, half-life≈7 months). Regular annual epidemics with an attack rate of around 15% are observed; antigenic diversity takes on the familiar pattern of gradual build-up followed by rapid collapse coinciding with extinction of prominent lineages; and phylogeny is generally linear over long time spans, with a moderate amount of short-term branching. **(D)** Alternative parameterization within the plausible region: moderate strength and intermediate duration strain-transcending immunity (strength≈63%, half-life≈28 months). As in part C, all measures are generally within acceptable ranges, and flu-like dynamics are reliably generated.

To understand how strength and duration of strain-transcending immunity affect outcomes, we begin by considering the behavior of the model within the regimes of extreme values: ineffective or perfect, ephemeral or permanent. The regime we consider first is the one closest to a model *without* strain-transcending immunity: ineffective and ephemeral (bottom-left in maps). Without the extra protection afforded by strain-transcending immunity, hosts are much more susceptible to repeated infection. This is reflected by a high incidence and a very high attack rate, although epidemic duration and reproductive number are within their target ranges. As a result of an increase in the number of infections, the virus has more opportunity to diversify, and we find that pairwise diversity and MRCA are particularly high, while Kappa and fixation rate are elevated within their target ranges. In this regime, we observe a weak selective pressure as minor antigenic changes are sufficient to evade specific immune responses. A characteristic trajectory within this parameter regime is shown in Fig 4A.

Perhaps the most extreme regime is that of perfect and permanent strain-transcending immunity (top-right in maps). Here we observe an antipodal response, both epidemiologically and evolutionarily. With such extreme protection, hosts are essentially protected for life following an initial exposure to the virus. As expected, extreme lows are reported for almost all measures. Epidemic duration, below its target but within its range, is the sole exception, presumably because the shape (though not the magnitude) of the epidemic curve remains on average unchanged. Due to a dearth of susceptible hosts, the virus is saved from stochastic extinction only by the eventual accumulation of newborn hosts in a manner reminiscent of measles. A characteristic trajectory within this parameter regime is shown in Fig 4B.

Next we consider two hybrid regimes of strain-transcending immunity: ineffective but permanent, and perfect but ephemeral (bottom-right and top-left in maps, respectively). We begin to observe reasonable outcomes for many measures in these regimes and find in particular that incidence and diversity are both satisfied within and between both regimes. According to most maps, the value being measured is relatively static throughout the transition from corner to corner. One exception to this trend is epidemic duration, which generally transitions from low to high as duration of strain-transcending immunity increases; another exception is fixation rate, which indicates very strong positive selection in the perfect-but-ephemeral regime and influenza-like positive selection in the ineffective-but-permanent regime. The latter result indicates that very short durations of strain-transcending immunity pressure the virus into a state of rapid, slight antigenic change, whereas very long durations of strain-transcending immunity pressure the virus into making less rapid, but more significant jumps in antigenicity. As the strength of strain-transcending immunity approaches zero, selective pressure depends only on adaptive immunity, which is, at least in the model, a nonlinear function of antigenic distance. As a result of this nonlinearity, rare large antigenic steps are more favorable than frequent small antigenic steps.

Finally we consider the plausible regime of strain-transcending immunity: effective (but imperfect) and of moderate duration (having a half-life on the order of several months to years). All eight measures are generally close to their target values when the model is parameterized within this regime, and we take an approach inspired by ABC to formally define the region of plausible parameterizations. By running simulations and contrasting model output with expected outcomes, we bypass explicit evaluation of a likelihood function while still generating a posterior distribution over parameterizations. We observe that while the default parameterization of strain-transcending immunity given in [4] is not rejected, it represents one extreme of the spectrum of plausible parameterizations of strain-transcending immunity, which extends much further in both dimensions, up to a half-life of 10 years at 50% strength at another extreme. Characteristic trajectories within this parameter regime are shown in Fig 4C and 4D.

The characteristic evolutionary trajectory of influenza has been studied in great depth, yet until individual hypotheses—including the existence and efficacy of strain-transcending immunity—are empirically tested, no single mechanism can be definitely posited as the driver of influenza’s evolution. Yet in many cases, such empirical validation remains infeasible for myriad reasons, including in particular the long time scales, large population and sample sizes, and prohibitive cost of biological measurements involved. Perhaps the largest obstacle facing empirical validation of strain-transcending immunity is the infeasibility of human testing. One of the simplest alternative approaches that has been applied herein and countless times in recent years has been to demonstrate the feasibility of particular hypotheses though mathematical and computational modeling. Such models, however, can only provide circumstantial support because a true empirical validation or rejection of a biological hypothesis requires biological evidence and experimentation. In this direction, a natural alternative approach to the validation of strain-transcending immunity in humans is to test for its existence in other mammals. Until now though, for the reasons mentioned above and others, such a study would be of a prohibitively large magnitude primarily due to the large span of durations and strengths of strain-transcending immunity to be tested. Here we describe much more precisely the biologically plausible ranges of strength and duration of strain-transcending immunity in the hope that future work toward its empirical validation can take advantage of the estimated ranges to greatly reduce the magnitude of the experimental search space.

Although it has yet to be conclusively shown that strain-transcending immunity is responsible for influenza’s characteristic epidemiological and evolutionary dynamics, there have been several recent studies providing empirical evidence that cellular immune responses are able to confer some degree of heterosubtypic immunity. In mice, cellular immunity has been shown to reduce illness [27] and protect against lethal infection following infection with different subtypes [28,29]. Similarly, cell-mediated heterologous immunity has been observed in ferrets [30,31]. Further, it has been suggested that weak heterologous immunity in humans is necessary to account for suppressed influenza B outbreaks following severe epidemics of influenza A [32].

Regarding the nature of antigenic evolution in the model (see Methods), we note that there are implicit constraints on the magnitude of antigenic change between viral progenitor and progeny strains which determine the process by which immune escape mutants arise. Antigenic evolution is concomitant with non-synonymous substitutions in viral codons, and although individual mutations can cause at most one amino acid substitution, mutations events are independent across nucleotides and can potentially occur simultaneously within an infected host on each day of infection. Although technically possible, it is exceedingly unlikely that any nascent mutant will differ from its parent strain at more than two amino acid sites, and because at least three amino acid substitutions are required to cross the immune escape threshold (in the current parameterization), any large antigenic changes will almost surely be the result of a series of mutation events over multiple days and across multiple hosts. While it has been demonstrated that models allowing for rare, but abrupt, changes in antigenicity are able to reproduce many of influenza’s characteristic dynamics without a need for strain-transcending immunity, the incremental changes in antigenicity modeled here are more consistent with our understanding of the empirical mechanisms of antigenic drift on the micro scale of short-term, within-host evolution [33]. However, on longer timescales it is generally understood that punctuated changes in antigenicity (which this model neither prescribes nor proscribes) drive the cluster transitions observed roughly every 2–5 years with influenza A/H3N2 [7,8,34].

Although the model successfully recapitulates the phylodynamics of influenza A/H3N2, it should be noted that there are certain design choices (see Methods) which complicate the interpretation of simulated trajectories. The current model assumes a universe consisting of only influenza A/H3N2, and is therefore unable to generate the seasonal variations in dominant subtypes observed empirically. Complicating the addition of these additional strain types is our limited understanding of the strengths and durations of the interactions between them. Another shortcoming of the present model is an absence of the effects of broad vaccination campaigns routinely used in many parts of the world. Mass vaccination against contemporary strains undoubtedly has a non-trivial effect on the shape of the epidemic trajectory which cannot be captured in the simplistic universe of the model. Additional improvements to the model could include using a more realistic population structure, perhaps taken from synthetic population estimates [35]; and incorporating climatological data, such as absolute humidity [36], to more accurately modulate transmissibility than the current sinusoidal forcing function. Finally, the simplistic, two-component immune system modeled here is a useful, but limited, abstraction of the complex and not entirely understood human immune response against rapidly evolving, antigenically variable viruses. For example, the confounding phenomena of original antigenic sin, antigenic seniority, antigen trapping, and back-boosting have a significant and non-trivial role in determining immune response [37,38], yet none of these effects are explicitly modeled. (Although the effects of transient strain-transcending immunity are arguably similar in some respects to the effects of back-boosting, which increases antibody titers against all previously encountered strains for a duration of time on the order of one year.) However, it would be straightforward to extend the model to emulate original antigenic sin and antigen trapping, and modeling the effect of back-boosting in a partially vaccinated population would be another interesting, although more challenging, direction for future work.

Beyond providing a more thorough characterization of strain-transcending immunity, we also contribute a methodology for evaluating simulated outcomes with respect to some set of reference outcomes. Determining whether and to what extent a model-generated trajectory matches expected outcomes for influenza has been a challenge for as long as influenza has been modeled, and a large number of individual metrics have been conceived and applied to the task. Unfortunately though, no standard methodology has been suggested for such evaluation, and outcomes from each model have generally been studied qualitatively with disjoint sets of measurements and methods. More problematic though is a lack of statistical support for these methods, in part because the measures employed are typically considered only in isolation. Here we show that many epidemiological and evolutionary measures can contribute simultaneously to our confidence in the plausibility of generated trajectories in comparison to a set of reference values. We posit and test the hypothesis that a simulation is plausibly influenza-like, enabling the assignment of likelihoods to individual trajectories and plausibility to specific parameterizations. Looking forward, we encourage the use of similar quantitative approaches to lend statistical support to analyses of simulated dynamics of infectious diseases.

## Models and Methods

**The model** given in [4] (the ‘Base Model’) is an individual-based generative model of the long-term spread and evolution of influenza within a human population. The reader is directed to the original publication for a complete reference, but for convenience the most salient details are recapitulated here. Individuals are distributed according to a semi-stochastic spatial hierarchy; they are assigned random coordinates drawn uniformly within a patch, with local clusters of hosts representing neighborhoods and the entire patch representing a large population center. Patches are arranged in a grid, and the top and bottom halves of the grid represent the northern and southern hemispheres, respectively. The likelihood of transmission between any pair of hosts is based on their proximity: within-neighborhood (R_0_ = 5), within-patch (R_0_ = 0.4), or cross-patch (R_0_≈0.004). A 25% sinusoidal seasonal forcing modulates transmissibility in opposite phase for the northern and southern hemispheres. Individual viral strains are defined by a small genome containing only the codons putatively under positive selection: 36 nucleotides encoding four hypothetical epitopes, each consisting of three amino acids. Antigenic phenotype is the set of 12 amino acids encoded by this genome, and under this parameterization there are 20^12^ possible distinct antigenic types. Following exposure to the virus, the probability of infection depends on the immune history of the exposed host. Immunity is conferred by two independent mechanisms: a long-term immunity against previously encountered strains and a short-term immunity against all strains. The long-term immunity provides very strong protection against strains that a host has been previously infected with and provides a weaker protection against strains that are similar to those known strains. In particular, the resistance afforded against a particular strain is a function of the number of novel amino acids encountered at each position, providing 99% resistance against strains with 2 or fewer novel amino acid substitutions (an immune escape threshold) and falling linearly to 25% resistance as the number of novel amino acid substitutions approaches 12. The short-term immunity provides a broad protection against all strains and decays exponentially over time (transient strain-transcending immunity). Upon infection, hosts incubate for two days, are infectious for four days, and are healthy afterward. When the host lifespan is reached, the host is respawned with an empty immune history. There is a small chance of mutation each day in each infected host (p = 1e-5 per nt), with new mutants arising (replacing the parent strain in the infected host) and going extinct stochastically.

**The simulator** is our implementation of the Base Model (see S1 Supporting Information), with the primary aim of studying how the parameterization of strain-transcending immunity affects the outcomes predicted by the model. Taking advantage of a considerable increase in available computing power over the past decade, we made several deviations from the default parameterization of the Base Model to relax some of the strong assumptions that were previously required. Foremost among these assumptions in [4] were a very small population size (default 12 million hosts) and a very short host lifespan (default 30 years). A direct consequence of a small population size combined with a long lifespan in the model is the stochastic extinction of the virus. Another potential problem that arises with such unrealistically small population sizes is that in some situations cross-immunity alone (lack of transient strain-transcending immunity) is able to constrain viral diversity [4,39]. Although the original study primarily used the smaller population size, it was demonstrated that reasonable outcomes could be obtained with a higher population size and longer lifespan, suggesting that the same dynamics could be reproduced under these conditions. Since we ran all simulations at the larger population size (100 million hosts), we were able to assume a more reasonable host lifespan of 60 years by reducing the volume of transmission between distinct geographical patches. These modest departures from the default parameterization of the Base Model give us more confidence that the model is a reasonable approximation of reality, and we expect that these changes will result in a more reliable set of outcomes.

**Plausibility** was determined by a chi-squared test as the target value and 95% credible range of each measure lead to a convenient definition of each target distribution. Assuming normally distributed target measures, we calculated the standard score for each simulated outcome; assuming that all measures are independent, we calculated the sum of squares of the eight standard scores to find the chi-squared test statistic at each simulated point in parameter space. We defined the plausibility region to be the set of grid points which were not rejected by a chi-squared test (df = 8, p > 0.05). Additional analysis relaxed the former assumption by leaving out measures with skewed target distributions and relaxed the latter assumption by estimating the covariance among measures and using a more accurate Mahalanobis distance. These additional analyses produce plausibility regions which are in agreement with the previously described results (see S1 Supporting Information).

## Supporting Information

S1 Supporting Information Measuring Outcomes, Simulator Details, Explored Parameter Space, Relaxing Assumptions, and Sensitivity Analysis. (PDF)

S1 Dataset Simulation data. Complete list of parameter strings, random seeds, final state hashes, and measured values for all 1200 simulations.
(CSV)
