# Supplementary material for: Computational Characterization of Transient Strain-Transcending Immunity against Influenza A
Source: PLoS One. 2015 May 1;10(5):e0125047. doi: 10.1371/journal.pone.0125047 (PMC4416895; doi:10.1371/journal.pone.0125047)
Supplement: S1 Supporting Information — (PDF) [file pone.0125047.s001.pdf]

Computational Characterization of Transient Strain-Transcending Immunity against Influenza A

David C Farrow, Donald S Burke, Roni Rosenfeld

Supporting Information

This document contains the following sections:

Methods: The eight measures and algorithms for their calculation; and simulator details.

Analysis: Parameter space, lifespan, tropics, and more stringent definitions of plausibility.

Data: Tabular recapitulations of main-text Figures 1-3 and SI Figure 2.

Methods

Text S1: Measuring Outcomes

Epidemiological measures (Annual Attack Rate (AAR), Epidemic Duration (ED), Reproductive Number (R_p), and Peak Weekly Incidence (PWI)) are determined solely from weekly incidence counts (Figure S1). The simulator records daily incidence in each patch, and weekly incidence is calculated (separately for each hemisphere) by summation of the daily incidence over each week and averaging across patches, and final incidence is reported in units of cases per 100,000. For each epidemic season (50 total), the four epidemiological measures are calculated. Finally, the measured values are averaged over all years, and over all simulation replicates (20 total).

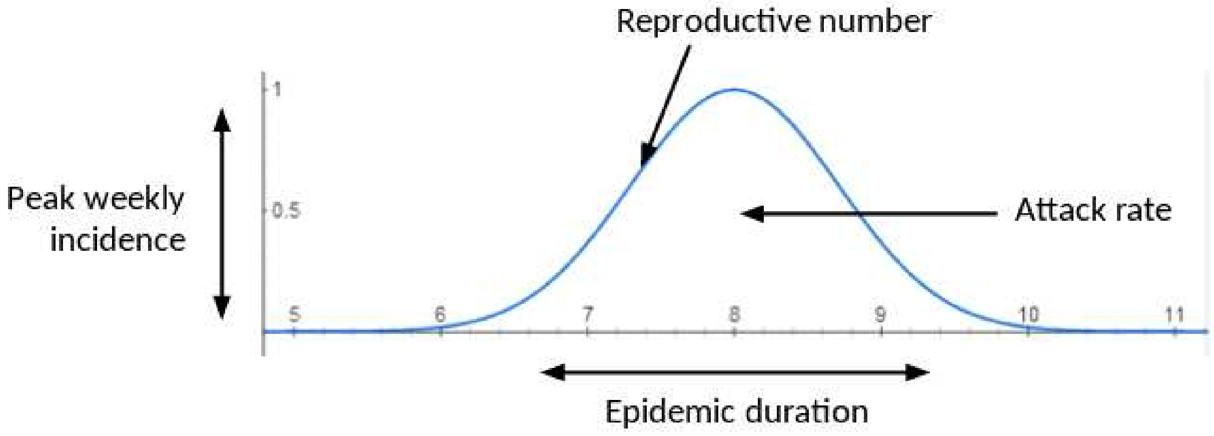

Figure S1. Visualization of epidemiological measures. A hypothetical weekly incidence curve is shown in blue. The x-axis represents time, and the y-axis represents incidence. The four epidemiological measures label the properties of the epidemic curve they capture.

- ➔ AAR: cumulative incidence per season.
- ➔ ED: number of weeks of sustained epidemic per season. Initially defined as 1 week (the single week of peak incidence), and expanded until 90% of the season's attack rate has been captured. Duration expands one week at a time, either forward or backward, whichever direction has a higher incidence (Listing S1).
- ➔ R_p: reproductive number of the virus per season, assuming a host population with lingering immunity from previous exposure. It is defined as the average ratio of incidence between consecutive weeks during the four weeks prior to the peak week. See [1].
- ➔ PWI: peak incidence per season.

```
start = end = <week of peak incidence>
sum = <peak incidence>
limit = <attack rate> * 0.9
while sum < limit:
        if(incidence[start - 1] > incidence[end + 1]):
                start = start - 1
                sum = sum + incidence[start]
        else:
                end = end + 1
                sum = sum + incidence[end]
ED = 1 + (end - start)
```

Listing S1. Pseudocode for calculating epidemic duration.

Evolutionary measures (Antigenic Diversity (AD), Fixation Rate (FR), Most Recent Common Ancestor (MRCA), and Kappa (κ)) are determined from viral peptide sequences (AD), nucleotide sequences (FR), and phylogeny (MRCA, κ). The simulator records peptide and nucleotide sequences for extant viruses on each day of simulation, and phylogeny is explicitly tracked throughout the course of the simulation.

- ➔ AD: average pairwise peptide sequence diversity (Hamming distance) between all extant strains at a given point in time. This is calculated each day of the simulation and averaged over all days. See [2].
- ➔ FR: average number of nucleotide substitutions per site per year. The simulator maintains a mutation counter for each extant virus, and the average number of mutations recorded for extant viruses at the end of the simulation is divided by the total number of years simulated. See [2].
- ➔ MRCA: the time since all extant viruses diverged from a common viral ancestor. Here we use an approximate definition: the average number of years of evolution separating two randomly sampled contemporaneous viruses (also known as π). See [3,4].
- ➔ κ : measure of antigenic diversification. This is defined as the expected number of additional surviving antigenic variants spawned per antigenic variant. See [5].

Text S2: Simulator Details

Parameters include all of those in the base model [2] and several others unique to our implementation (Table S1). In general, default values were taken from the base model except for (1) increasing the host lifespan, (2) increasing the population size, and (3) sweeping over strain-transcending immunity parameter space. To increase host lifespan, the *hostLifespan* parameter was changed from 30 years to 60 years. To increase the population size, the *hostsPerPatch* parameter was changed from 600,000 to 5,000,000. Ferguson et al. also simulated a larger population size under a slightly different connectivity structure and noted that the coupling between patches needed to be reduced to generate realistic outcomes; here we reduced that coupling (via the *R0_global* parameter) from 0.02 to 0.00368. Finally, we explore the parameter space of strain-transcending immunity as described before by varying the omega and tau parameters, representing the strength and timescale of strain-transcending immunity respectively.

The burn-in period was simulated once; a canonical 100-year burn-in simulation was run under the default parameterization, and the simulator state was saved afterwards. All remaining simulations were 50-year continuations, under various parameterizations, of this saved state.

The population was generated as described by [2]. The population consists of a set of patches, and each patch is generated by the following process. A number of hosts (*hostsPerPatch*) are spawned with (x, y) coordinates drawn uniformly at random. The average minimum distance between all pairs of hosts is measured, and all host coordinates are rescaled so that the resulting average minimum separation between all pairs of hosts is 1 unit. Finally, the *neighborhoodRadius* parameter is used to determine, for each host, which other hosts are within its local neighborhood. We find that on average each neighborhood contains roughly 12.55 hosts.

Source code is publicly available on GitHub as two complementary repositories: a cross-platform Java implementation (https://github.com/undefx/FM_Java) and a faster (but only built on linux) C++ implementation (https://github.com/undefx/FM_Cpp). Given the same initial parameterizations, both implementations will generate deterministic, bit-identical outcomes. Population files must be generated with FM_Java, but can be used with both FM_Java and FM_Cpp.

Table S1. Simulator parameters and default values.

Name	Default Value	Purpose
omega*	1	Strength of strain-transcending immunity.
tau*	270 days	Timescale of strain-transcending immunity; 6-month half-life.
theta0	25%	Minimum strength strain-specific immunity.
theta1	99%	Maximum strength strain-specific immunity.
nt	2	Number of mutations after which strain-specific immunity declines.
seasonalityMultiplier	25%	Amplitude of sinusoidal seasonal forcing.
neighborhoodRadius	4	The radius of a neighborhood.
mutationProbability	1e-5 per nt	The per-nucleotide probability of mutation.
numEpitopes	4	Number of epitopes.
codonsPerEpitope	3	Number of codons per epitope.
numPatches	20	Number of patches.
hostsPerPatch*	5,000,000	Number of hosts per patch; 100 million total hosts by default.
hostLifespan*	21900 (60 years)	The host lifespan.
R0_local	5	Within-neighborhood R_0.
R0_patch	0.4	Within-patch R_0.
R0_global*	0.00368	Between-patch R_0.
numDays	18250 (50 years)	The number of days to simulate.
minCarriers	1	A number of hosts to remain infected to prevent stochastic extinction of the virus.

* Default value changed.

Reproduction of the results reported herein is straightforward, assuming appropriate hardware is available. The simulator is CPU and memory intensive, and at least 32 GB of RAM is recommended. After compiling the simulator (assuming the Java version is being used), the first step is to generate a population file. The random seed used for the simulations in this study was 0x123abc (1194684 decimal). The total population size is 100 million hosts, but these are distributed evenly among 20 patches of 5 million hosts. The population file can be generated with the following command:

```
java -jar FM_Java.jar -patch 1194684 5000000
```

The outfile file, named “patch-5000000.bin” will have an MD5 hash of 57a831d1dd1d8c66e6fc20525af3badb. The next step is to simulate the initial 100-year burnin period. The purpose of this genesis simulation is to allow the system time to equilibrate under the default parameterization of the [2] model. At the end of this initial 100-year simulation, the simulator will dump it’s state to disk for further processing. To run the simulator, a compressed parameter string (also called the data string) is required. For the particular genesis simulation used here, the random seed was 0x12345 (74565 decimal), and the other parameters were set according to Table S1. The following command can be used to run the genesis simulation and generate the requisite saved state file for subsequent simulations:

```
java -jar FM_Java.jar -new eJxjZCAXpNg5aYS11pCsj1HZFYULAKqtAs8=
```

After the simulation finishes, the simulator will generate a 64-bit final state hash to uniquely identify the simulated trajectory. The value of this hash will be 906e89e62c5def76 for the genesis simulation described above. The output file will be named according to the following pattern: “stats-#.bin”, where # is the current system time in milliseconds. For the sake of convenience later on, the file can be renamed to, for example, “genesis.bin”. At this point, the 1200 individual 50-year simulations can be run. To repeat any simulation, lookup its data string (see the supplementary file “S1_Dataset.csv”, Dataset S1) and instruct the simulator to resume the genesis simulation with the new set of parameters. The command, using as an example the first simulation listed, would be:

```
java -jar FM_Java.jar -resume genesis.bin
eJxjZMAFFKC0bQJ2+RQ7J42w1hqc+pHBr/jUzRmfFjIwMDq+0qgFCpy8B5cDAIoXCwY=
```

The final state of the simulation executed by the above command will be 33db064d66af386e. The simulator includes an API to read the generated output file, and the algorithms described in Text S1 can be used to calculate the value of each of the 8 measures for any given simulation (these values are also provided in the supplementary file "S1_Dataset.csv", Dataset S1). Table S2 contains, among other things, the mean and sample standard deviation of these values grouped by parameterization (n=20 for each set of parameters).

Analysis

Text S3: Explored Parameter Space

The aim of this study is to explore all computationally practical, non-trivial parameterizations of strain-transcending immunity and to then suggest which of those are also likely to be biologically plausible. Because this parameter space is continuous (and extends infinitely in the half-life dimension), it is necessary to place some reasonable bounds on the exploration space and to sample a discrete set of parameterizations within those bounds. In the limits of strength approaching zero efficacy or half-life approaching zero time, no protection is conferred by strain-transcending immunity; in the limit of exceedingly long half-life at any appreciable strength, strain-transcending immunity degenerates into a static reduction in susceptibility [2]. Pragmatically, we bounded the primary exploration to strengths within [0.25, 1.00] and half-lives within [41 days, 60 years]. Additional simulations (not shown) indicate that only extremely implausible parameterizations lie outside of these bounds. The discrete grid of 60 points that we imposed on the bounded region is linear in strength of strain-transcending immunity and exponential in half-life of strain-transcending immunity. The exponential step-up in half-life was chosen to satisfy two constraints: selecting a computationally practical number of grid points while sufficiently sampling each regime.

Text S4: Relaxing Assumptions

Throughout the main-text and supporting information, plausibility at a particular point in parameter space is determined by (1) measuring the Mahalanobis distance (MD) from averaged measures taken on model output to the empirically derived targets for influenza and (2) testing the null hypothesis that the point is not a multivariate outlier. If the hypothesis is rejected (at the α=0.05 level), then the parameterization is deemed implausible; otherwise, the point is considered to be plausible. In the main-text, two simplifying assumptions are made: that empirical targets are normally distributed and that all measures are independent (Figures 1 and 2; Table S3). Here, we relax both assumptions and show that outcomes in terms of plausibility are largely unaffected by these assumptions.

To relax the assumption that the empirical targets ranges are normally distributed for all measures, we exclude the two of the eight measures which are explicitly not normally distributed: MRCA and κ. We repeat the process of establishing plausibility by now comparing Mahalanobis distance to a chi-squared distribution with six degrees of freedom (Figure S2, Table S3). We observe only minor changes to the plausibility region and conclude again that the intermediate regime of strain-transcending immunity is plausible.

Seperately, we relax the assumption of independence among measures. We take two approaches: (1) excluding measures which are suspected to be highly correlated, and (2) using an estimated covariance matrix to calculate MD. Regarding the first approach, we exclude two presumably highly correlated measures: PWI and AD. We again compare Mahalanobis distance to a chi-squared distribution with six degrees of freedom (Figure S2, Table S3) and observe only minor variation in the shape of the plausibility region.

- ➔ Of the PWI (incidence, *height*), ED (duration, *width*) AAR (attack rate, *area*) triplet, any individual measure can be reasonably estimated given the other two, assuming there is some canonical epidemic shape. We exclude PWI because we are more confident in the target distributions of ED and AAR than of PWI.
- ➔ AD (diversity) and MRCA (common ancestor) both essentially measure the amount of divergence among extant strains. Given a distant MRCA, high diversity is not surprising; given a recent MRCA, low diversity is not surprising (and *vice versa*). We exclude AD because *true* MRCA can be reasonably estimated from empirical sequence data, whereas it is not clear how to estimate *true* AD from empirical sequence data.

Regarding the second approach, we first estimate the sample correlation among measures. We generate this estimate separately at each grid point (recall that n = 20 simulations at each point) and observe that much of the correlation structure is preserved across parameter space. We illustrate this correlation structure below, showing the average of all individual correlation matrices (Figure S3). Until now we implicitly employed a diagonal covariance matrix when calculating MD, where diagonal elements were variances and non-diagonal elements were zero (no correlation between measures). Now, we build a more complex covariance matrix by using simulator output to

estimate correlations between measures. We generate each covariance matrix separately at each grid point by (1) converting the estimated correlation matrix into a covariance matrix (scaling by standard deviations of target values across rows and columns) and (2) element-wise averaging the old (diagonal) and new (non-diagonal) covariance matrices (diagonals are equal; non-diagonals take 50% of the correlation structure to avoid overfitting). We use the resulting covariance matrices to calculate a MD which takes into account correlations between measures (Figure S2, Table S3). Again, the shape of the plausible region remains essentially unchanged.

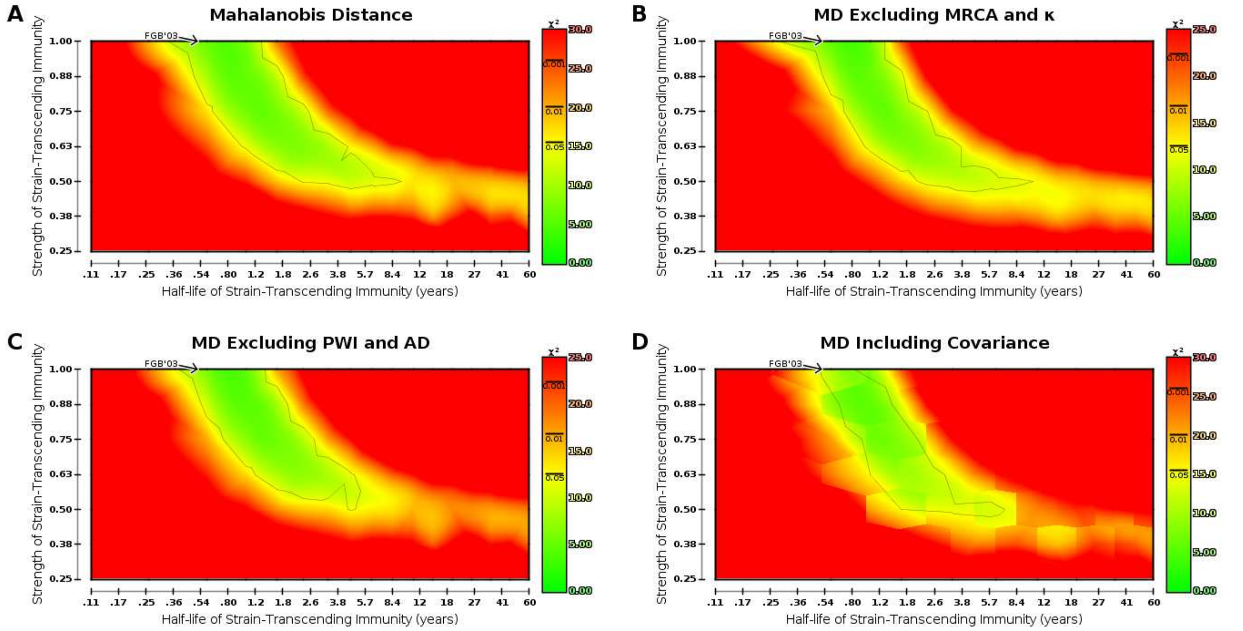

Figure S2. Plausibility, excluding MRCA and Kappa. Maps are as described main-text Figure 3. Mahalanobis distance is compared to a chi-squared distribution to reject parameterizations which result in multivariate outliers. **(A)** Original plausibility map from main-text Figure 3, repeated here for convenience. **(B)** Excluding MRCA and κ (not normally distributed); χ^2 df=6. **(C)** Excluding PWI and AD (highly correlated with other measures); χ^2 df=6. **(D)** Estimating correlations and using a non-diagonal covariance matrix at each point; χ^2 df=8. (Per-pixel covariance matrix set to nearest grid point covariance matrix.)

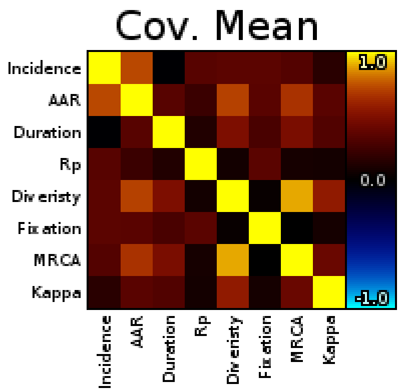

Figure S3. Average of 60 sample correlation matrices. The strongest correlations are on average between MRCA and AD (diversity): 0.90; AAR and PWI (incidence): 0.73; AAR and AD (diversity): 0.71; and AAR and MRCA: 0.67.

Host Lifespan

Global life expectancy is on the order of 60-70 years [6], and this is the value that we matched in our simulations.

However, because the simulated population is unable to accommodate a variable (increasing) number of hosts, it is also interesting to simulate a lifespan that is closer to the global median age, which is on the order of 30 years [7]. To determine to what extent shorter host lifespans affect our results regarding the plausibility of transient strain-transcending immunity, we (1) held the immunity parameters constant (at a strength of 87.5% and half-life of 1 year) while varying host lifespan from 30-60 years and (2) remapped a large portion of parameter space under a host lifespan of 30 years. For the first sensitivity analysis of host lifespan (constant immunity parameters, variable lifespan), we ran sets of at least 10 replicates at 30, 40, 50, and 60 year lifespans and measured median Mahalanobis distance for each set (Figure S4). Although the distance increases slightly as the host lifespan is decreased, all of the parameterizations remain well within the plausible range; a distance of 15.51 or greater is required to reject a hypothesis using a chi-squared distribution with 8 degrees of freedom.

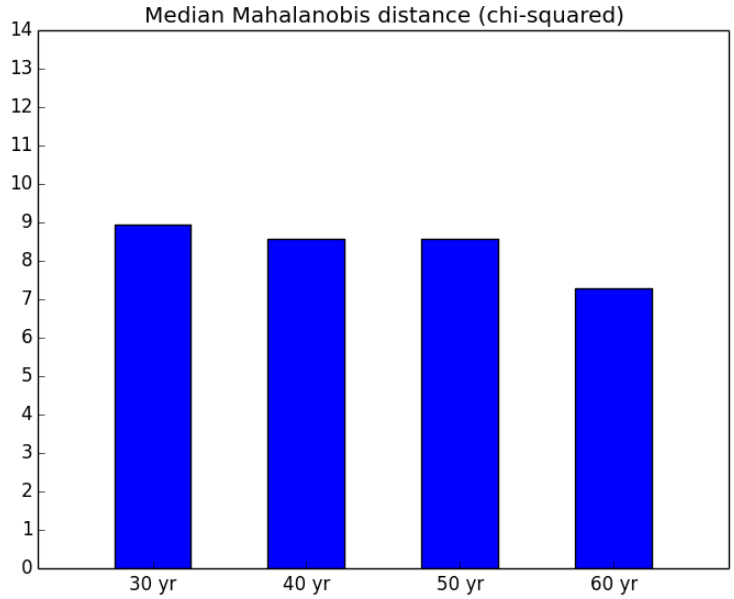

Figure S4. Sweeping host lifespan while holding all other parameters constant. Median Mahalanobis distance is plotted for four sets of lifespans. (n=10 for 30, 40, and 50 years; n=20 for 60 years).

For the second sensitivity analysis of host lifespan (constant 30-year lifespan, variable immunity parameters), we ran sets of at least 5 replicates across the most salient portion of parameter space to determine how the shape of the plausible region is affected (Figure S5). Although the shape of the plausible region changes slightly, we still find that our main conclusion - that it is possible that transient strain-transcending immunity could be weaker and longer lasting than previously thought - still holds. As expected, reducing lifespan (equivalently, increasing respawn frequency) results in an overall increase in the number of naive hosts, and attack rates are uniformly, but not unreasonably, increased compared to those of the simulations using 60 year lifespans (shifting the region of acceptable attack rates to the right on the map; Figure S6).

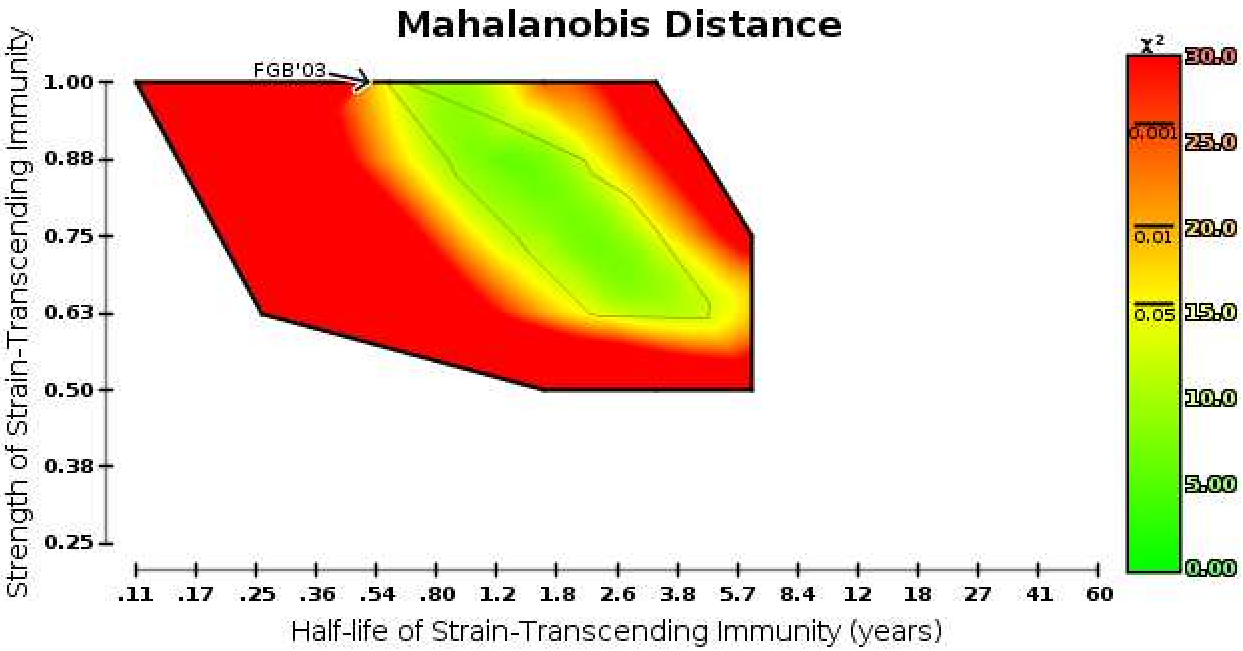

Figure S5. Plausibility map using a reduced host lifespan of 30 years. Map illustrates rejection of immunity parameterizations as previously described. Although the plausible region is smaller, the shape is essentially unchanged; immunity half-lives on the order of years are plausible with a reduction in strength. (n=5 at all points, except the point used in the lifespan sweep described above, for which n=10.)

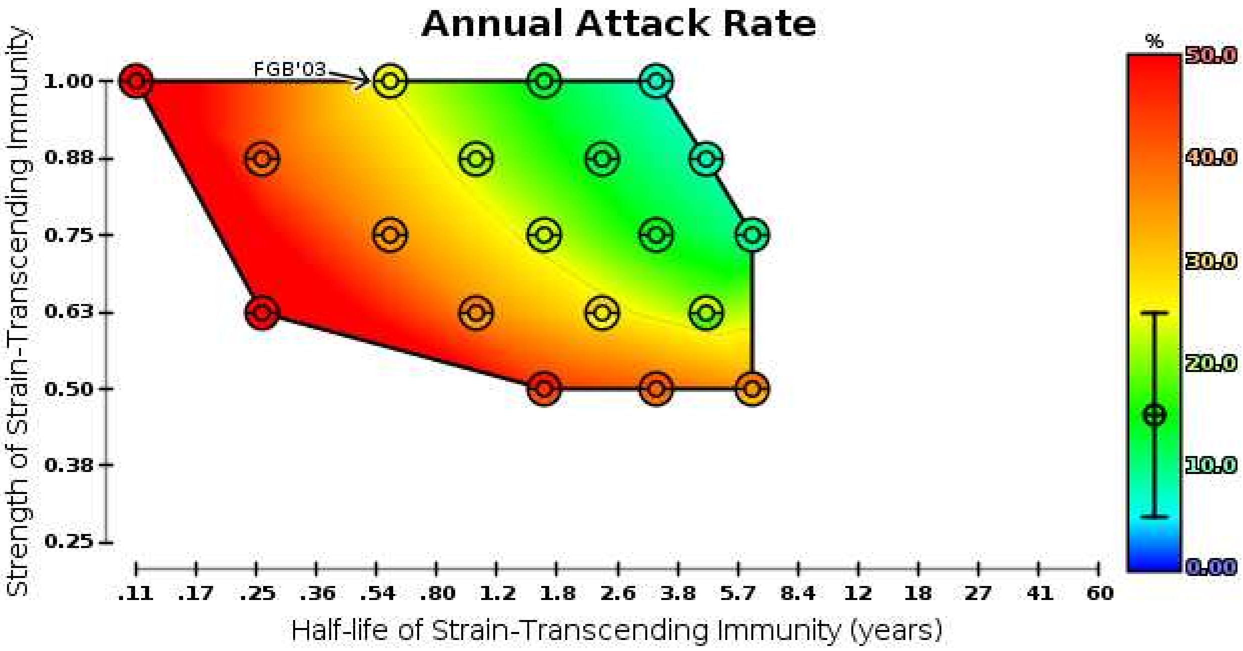

Figure S6. Annual attack rate map using a reduced host lifespan of 30 years. Map is as in main text Figure 1A. Reduced lifespan results in more frequent host respawning and a more rapid increase in the number of naive hosts. Although attack rates are correspondingly higher across parameter space, they are not always unreasonably high. (n=5 at all points, except the point used in the lifespan sweep described above, for which n=10.)

Inclusion of a Tropical Deme

The simple world of the model consists of two equally populated hemispheres which are subjected to sinusoidal seasonal forcing in opposite phase. In the real world, seasonal forcing is much more complex, and only the temperate regions of the world experience the type of seasonal forcing that can be described by a simple sinusoid with an annual peak in the winter months. The highly-populated tropical regions of the world are not subject to such well-defined forcing, yet it has been shown that strains arising and circulating in these tropical regions play a significant role in the diversification and spread of flu globally [8,9]. It is therefore of great interest what impact the simple geography of the model has on immunity results and how a more realistic model of the world would change those results. To this end we made a straightforward extension to the model to allow for patches representing tropical regions. These patches differ from the temperate patches only in that there is no imposed seasonal forcing. Additionally we redistributed the 20 patches (originally divided into 10 northern and 10 southern patches) into 8 northern, 10 tropical, and 2 southern patches to more closely approximate the actual distribution of the world population. All other aspects of the model, including between-patch contact rates, remained the same. As with the sensitivity analysis of host lifespan, we ran sets of 5 replicates across a broad range of parameter space to assess the impact of including tropical dynamics on the shape of the plausible region of immune parameters (Figure S7). We find that the shape of the plausible region changes somewhat more significantly, in particular becoming more jagged (presumably an artifact of a small number of replicates), and more importantly only extending up to a half-life of 2 years. Despite these moderate changes to the shape of the plausible region under a remarkably different model of the world, our conclusion remains in essence the same: transient strain-transcending immunity could plausibly persist with a half-life meaningfully longer than originally anticipated.

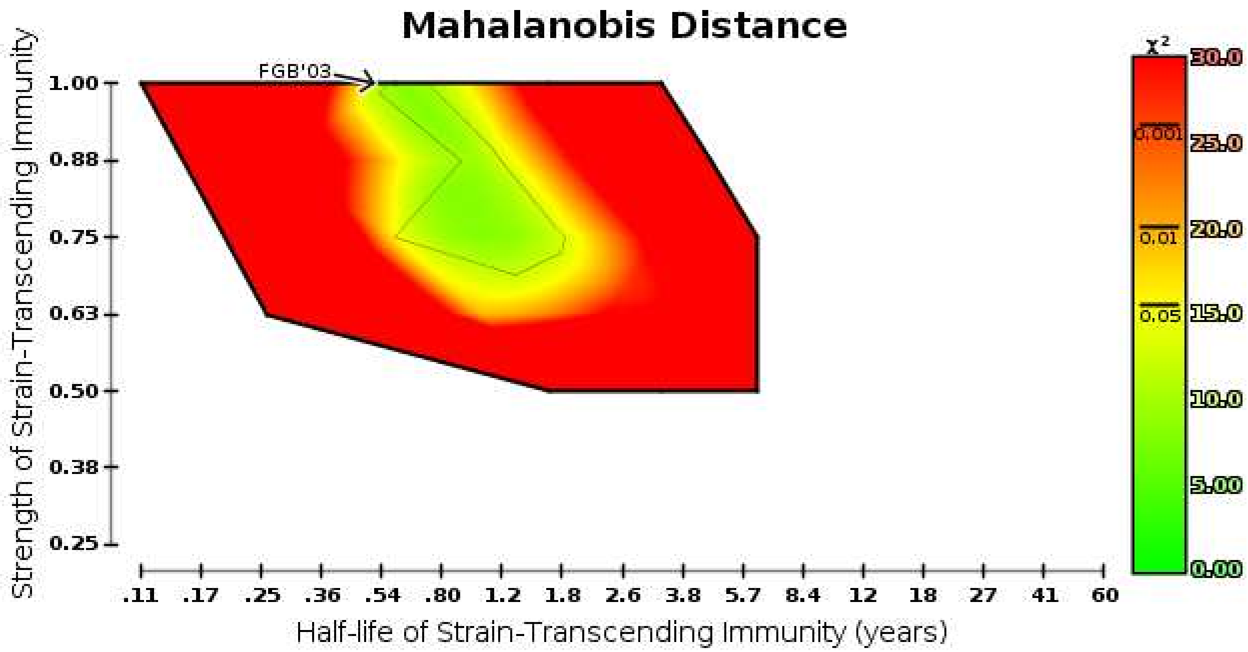

Figure S7. Plausibility map with the world including a large tropical deme. Map illustrates rejection of immunity parameterizations as previously described. Although the shape of the plausibility region has been perturbed, the general location of the plausible region remains unchanged. Immunity half-lives of up to two years are still plausible with a reduction in strength. (n=5 at all points.)

Data

The tables below contain the values which were used to generate the figures in the main-text. The complete list of parameter strings, random seeds, final state hashes, and measured values for all 1200 main-text simulations are available in the supplementary data file “S1_Dataset.csv” (S1 Dataset).

References

1. Chowell G, Miller Ma, Viboud C (2008) “Seasonal influenza in the United States, France, and Australia: transmission and prospects for control.” *Epidemiology and infection* 136: 852-64
2. Ferguson NM, Galvani AP, Bush RM (2003) “Ecological and immunological determinants of influenza evolution.” *Nature* 422: 428-33
3. Bedford T, Rambaut A, Pascual M (2012) “Canalization of the evolutionary trajectory of the human influenza virus.” *BMC biology* 10: 38
4. Zinder D, Bedford T, Gupta S, Pascual M (2013) “The roles of competition and mutation in shaping antigenic and genetic diversity in influenza.” *PLoS pathogens* 9: e1003104
5. Koelle K, Ratmann O, Rasmussen Da, Pasour V, Mattingly J (2011) “A dimensionless number for understanding the evolutionary dynamics of antigenically variable RNA viruses.” *Proceedings. Biological sciences / The Royal Society* 278: 3723-30
6. http://www.who.int/gho/mortality_burden_disease/life_tables/en/
7. https://www.cia.gov/library/publications/the-world-factbook/fields/2177.html
8. Russell C, Jones T, Barr I, Cox N, Garten R, et al. (2008) The global circulation of seasonal influenza A (H3N2) viruses. Science (New York, N.Y.) 320: 340-6
9. Bedford T, Rambaut A, Pascual M (2012) Canalization of the evolutionary trajectory of the human influenza virus. BMC biology 10: 38

Table S2. Epidemiological and evolutionary parsimony measured across strain-transcending immunity parameter space.

Half-life is shown in years; other units are as described in the main text. n=20 for all rows.

Parameterization		Sample Mean								Sample Standard Deviation							
Half-life	Strength	PWI	AAR	ED	Rp	AD	FR	MRCA	κ	PWI	AAR	ED	Rp	AD	FR	MRCA	κ
0.1	0.25	4.151	51.556	13.252	1.172	3.465	0.060	4.042	0.445	0.2800	2.7471	0.8611	0.0197	0.6680	0.0071	1.9382	0.0819
0.1	0.50	3.812	45.977	12.732	1.200	3.323	0.059	3.452	0.432	0.2524	1.7317	0.5972	0.0158	0.3601	0.0054	0.8495	0.0483
0.1	0.75	3.524	41.051	12.077	1.218	3.085	0.063	3.112	0.482	0.2515	1.4274	0.8359	0.0208	0.4104	0.0057	1.1188	0.0739
0.1	1.00	3.287	36.671	11.446	1.244	2.794	0.067	2.634	0.441	0.1580	0.9719	0.7545	0.0117	0.2104	0.0051	0.7245	0.1028
0.3	0.38	4.347	46.154	11.797	1.229	2.918	0.065	2.552	0.407	0.2182	1.5132	0.5920	0.0257	0.3533	0.0055	0.7055	0.0922
0.3	0.63	4.097	38.697	9.947	1.256	2.473	0.067	1.792	0.404	0.1655	1.0940	0.4478	0.0285	0.2351	0.0055	0.3573	0.1043
0.3	0.88	3.459	32.076	9.032	1.276	2.172	0.069	1.483	0.376	0.1224	0.7974	0.3568	0.0094	0.1924	0.0045	0.2644	0.0923
0.6	0.25	4.305	46.998	12.504	1.208	2.875	0.064	2.523	0.425	0.3071	2.5330	0.6281	0.0234	0.4184	0.0063	0.8246	0.0845
0.6	0.50	3.938	37.843	11.015	1.239	2.435	0.066	1.884	0.372	0.1524	1.4089	0.3064	0.0150	0.3934	0.0058	0.6825	0.0844
0.6	0.75	2.948	28.684	11.157	1.232	2.059	0.066	1.439	0.360	0.0935	0.7575	0.2416	0.0053	0.2184	0.0041	0.2477	0.0748
0.6	1.00	2.004	21.003	11.573	1.209	1.818	0.060	1.417	0.388	0.0604	0.4210	0.1959	0.0055	0.1972	0.0034	0.2616	0.0949
1.0	0.38	3.952	41.225	12.312	1.204	2.929	0.062	2.693	0.407	0.2052	1.9698	0.4387	0.0168	0.4906	0.0051	0.9395	0.1083
1.0	0.63	2.783	28.470	12.369	1.204	2.187	0.061	1.634	0.434	0.1007	0.9121	0.1759	0.0104	0.2611	0.0036	0.3414	0.1066
1.0	0.88	1.705	18.642	13.157	1.181	1.658	0.056	1.413	0.354	0.0577	0.4671	0.2143	0.0075	0.1803	0.0031	0.2309	0.0813
1.6	0.25	4.114	44.458	12.669	1.190	2.847	0.062	2.465	0.446	0.2502	2.1416	0.5474	0.0296	0.4405	0.0043	0.9300	0.0955
1.6	0.50	2.972	30.652	12.909	1.197	2.252	0.061	1.835	0.368	0.1569	1.7656	0.3316	0.0160	0.3637	0.0030	0.6563	0.0763
1.6	0.75	1.685	17.973	13.760	1.175	1.605	0.052	1.356	0.340	0.0504	0.7015	0.2357	0.0135	0.1880	0.0031	0.1892	0.0845
1.6	1.00	0.963	11.100	14.218	1.134	1.277	0.041	1.345	0.455	0.0333	0.3381	0.2957	0.0182	0.1773	0.0021	0.1637	0.1465
2.4	0.38	3.386	36.140	13.108	1.181	2.524	0.059	2.328	0.384	0.2012	2.4175	0.3967	0.0272	0.4724	0.0047	1.0332	0.1425
2.4	0.63	1.899	20.273	13.872	1.172	1.731	0.052	1.433	0.351	0.0993	1.3500	0.4545	0.0212	0.3031	0.0036	0.3437	0.1335
2.4	0.88	0.964	10.412	14.260	1.142	1.114	0.037	1.176	0.325	0.0307	0.3881	0.4998	0.0303	0.1544	0.0031	0.1576	0.1436
3.3	0.25	3.886	43.065	13.350	1.181	2.792	0.062	2.424	0.445	0.2095	1.7204	0.3989	0.0213	0.3030	0.0059	0.7651	0.0835
3.3	0.50	2.451	26.361	13.784	1.168	2.162	0.054	1.879	0.383	0.1654	2.0455	0.3788	0.0162	0.4798	0.0040	0.7857	0.1085
3.3	0.75	1.062	11.439	14.158	1.140	1.083	0.038	1.063	0.240	0.0486	0.6614	0.5700	0.0361	0.2149	0.0038	0.2104	0.0958
3.3	1.00	0.537	6.001	13.630	1.069	0.668	0.023	0.883	0.229	0.0268	0.2488	0.6836	0.0439	0.1634	0.0019	0.1994	0.1505
4.6	0.38	3.102	33.565	13.504	1.170	2.463	0.056	2.102	0.425	0.2385	3.3565	0.6190	0.0241	0.5899	0.0051	0.8697	0.1350
4.6	0.63	1.349	14.653	14.473	1.149	1.304	0.044	1.160	0.298	0.0648	0.7757	0.4211	0.0266	0.2638	0.0027	0.2790	0.1463
4.6	0.88	0.568	6.524	14.046	1.065	0.750	0.026	0.938	0.265	0.0153	0.3434	0.5734	0.0340	0.1802	0.0022	0.2561	0.1727
6.3	0.25	3.720	41.682	13.452	1.166	2.822	0.059	3.110	0.394	0.2585	3.2156	0.6844	0.0327	0.6220	0.0058	1.6276	0.0817
6.3	0.50	2.072	22.745	14.368	1.156	2.010	0.050	1.730	0.380	0.1356	1.8232	0.4457	0.0244	0.3845	0.0055	0.5839	0.1048
6.3	0.75	0.686	7.773	14.111	1.087	0.893	0.028	1.001	0.284	0.0393	0.6321	0.8630	0.0469	0.2124	0.0031	0.2582	0.1507
6.3	1.00	0.326	3.808	13.335	0.988	0.451	0.015	0.727	0.212	0.0146	0.2190	1.0104	0.0503	0.1500	0.0018	0.2715	0.1827
8.5	0.38	2.922	32.318	13.872	1.154	2.494	0.055	2.259	0.417	0.1627	2.1552	0.5627	0.0292	0.3945	0.0038	0.7873	0.1051
8.5	0.63	1.017	11.521	14.268	1.122	1.150	0.036	1.075	0.322	0.0632	0.8929	0.6401	0.0324	0.1759	0.0030	0.1956	0.1173
8.5	0.88	0.377	4.357	13.635	1.011	0.528	0.017	0.765	0.229	0.0243	0.2845	0.8231	0.0519	0.1539	0.0024	0.2468	0.1531
11.3	0.25	3.740	42.443	13.568	1.165	2.892	0.058	2.892	0.440	0.2179	2.3581	0.6802	0.0257	0.5076	0.0047	1.1192	0.0812
11.3	0.50	1.841	20.872	14.578	1.139	1.969	0.047	1.804	0.412	0.1733	2.3676	0.4925	0.0228	0.4595	0.0038	0.7675	0.0894
11.3	0.75	0.482	5.603	13.911	1.041	0.628	0.021	0.806	0.227	0.0339	0.4846	0.7393	0.0647	0.1654	0.0026	0.2376	0.1576
11.3	1.00	0.220	2.594	12.356	0.896	0.275	0.010	0.503	0.115	0.0134	0.1483	0.7239	0.0537	0.1090	0.0018	0.2159	0.1310
15.1	0.38	2.717	29.803	13.975	1.153	2.212	0.053	1.886	0.435	0.1732	2.5163	0.5925	0.0388	0.5159	0.0049	0.8195	0.1317
15.1	0.63	0.839	9.706	14.651	1.103	1.087	0.031	1.119	0.318	0.0847	1.0693	0.8199	0.0416	0.3258	0.0040	0.3751	0.1392
15.1	0.88	0.263	3.094	13.236	0.936	0.341	0.012	0.550	0.132	0.0153	0.2095	0.6453	0.0621	0.0810	0.0019	0.1356	0.0805
20.0	0.25	3.676	41.209	13.596	1.159	2.841	0.059	2.695	0.456	0.2340	2.7083	0.4608	0.0417	0.3943	0.0042	1.0327	0.0992
20.0	0.50	1.699	19.381	14.735	1.128	1.871	0.044	1.886	0.367	0.1526	2.2484	0.8119	0.0239	0.5847	0.0033	1.0925	0.1504
20.0	0.75	0.377	4.406	13.786	1.011	0.499	0.017	0.684	0.161	0.0182	0.2590	0.6111	0.0446	0.1587	0.0022	0.1827	0.1256
20.0	1.00	0.165	1.886	11.137	0.791	0.142	0.006	0.266	0.045	0.0136	0.0562	0.7867	0.0691	0.0753	0.0011	0.1490	0.0907
26.4	0.38	2.746	31.389	14.119	1.148	2.602	0.052	2.444	0.456	0.1948	2.2401	0.5277	0.0326	0.4298	0.0053	0.9092	0.1017
26.4	0.63	0.666	7.882	14.498	1.054	0.850	0.028	0.924	0.244	0.0581	0.8261	0.8579	0.0430	0.2032	0.0036	0.2614	0.1209
26.4	0.88	0.198	2.265	12.284	0.855	0.170	0.008	0.274	0.015	0.0163	0.1942	0.5723	0.0591	0.0524	0.0019	0.0707	0.0250
34.7	0.25	3.663	41.159	13.502	1.156	2.722	0.058	2.462	0.402	0.2543	2.2678	0.6934	0.0329	0.3825	0.0041	0.9242	0.0733
34.7	0.50	1.526	17.398	14.530	1.110	1.642	0.041	1.522	0.364	0.1409	2.2439	0.5636	0.0527	0.4674	0.0038	0.7158	0.1522
34.7	0.75	0.294	3.433	13.340	0.953	0.377	0.013	0.530	0.104	0.0236	0.2517	0.6581	0.0721	0.1102	0.0024	0.1622	0.0674
34.7	1.00	0.129	1.510	11.109	0.767	0.079	0.004	0.202	0.023	0.0143	0.0783	0.6387	0.0577	0.0440	0.0015	0.0931	0.0496
45.7	0.38	2.733	30.814	13.981	1.147	2.398	0.052	2.225	0.418	0.2459	3.3638	0.7315	0.0400	0.5832	0.0047	1.1054	0.0931
45.7	0.63	0.572	6.730	14.283	1.056	0.695	0.024	0.773	0.192	0.0636	0.8804	0.6381	0.0595	0.1880	0.0033	0.2057	0.1125
45.7	0.88	0.166	1.930	12.064	0.835	0.149	0.006	0.265	0.023	0.0155	0.1233	0.9004	0.0808	0.0741	0.0015	0.1092	0.0475
60.0	0.25	3.652	41.042	13.341	1.145	2.676	0.058	2.355	0.429	0.2469	3.2112	0.6495	0.0456	0.4449	0.0051	0.8660	0.1256
60.0	0.50	1.430	16.367	14.526	1.101	1.572	0.040	1.454	0.360	0.2338	3.1332	0.6700	0.0425	0.4928	0.0056	0.7354	0.1478
60.0	0.75	0.248	2.953	13.284	0.922	0.319	0.011	0.499	0.143	0.0202	0.3820	0.9483	0.0626	0.1246	0.0031	0.2212	0.1437
60.0	1.00	0.112	1.354	10.885	0.738	0.039	0.001	0.156	0.009	0.0074	0.0552	0.8696	0.0731	0.0174	0.0012	0.0635	0.0177

Table S3. Mahalanobis distance measured across strain-transcending immunity parameter space.

Half-life is shown in years; strength is dimensionless; other units are Mahalanobis distances. n=20 for all rows. *Not rejected (p > 0.05), shaded green.

Parameterization		Mahalanobis Distance			
Half-life	Strength	Main Fig. 3	SI 2B	SI 2C	SI 2D
0.1	0.25	137.57	71.53	124.14	129.27
0.1	0.50	92.97	51.42	82.91	79.55
0.1	0.75	70.07	38.39	63.51	60.84
0.1	1.00	48.70	30.87	45.07	59.90
0.3	0.38	69.78	54.56	60.34	74.64
0.3	0.63	44.00	40.14	38.57	42.35
0.3	0.88	33.98	32.52	32.23	36.33
0.6	0.25	70.77	55.75	61.92	63.66
0.6	0.50	37.50	33.41	33.06	43.03
0.6	0.75	16.86	15.79	16.49	20.88
0.6	1.00	6.47*	4.98*	5.90*	10.90*
1.0	0.38	57.33	38.97	50.13	56.31
1.0	0.63	14.58*	11.16*	14.29	15.53
1.0	0.88	6.15*	5.24*	4.55*	6.02*
1.6	0.25	61.50	47.11	54.00	47.07
1.6	0.50	18.46	14.90	17.81	15.52
1.6	0.75	7.48*	6.86*	5.67*	8.55*
1.6	1.00	23.29	20.33	17.00	25.02
2.4	0.38	34.88	24.45	32.39	31.67
2.4	0.63	8.14*	7.23*	7.21*	11.53*
2.4	0.88	26.49	26.02	19.16	30.46
3.3	0.25	57.48	43.91	51.56	54.57
3.3	0.50	14.49*	10.24*	14.38	14.32*
3.3	0.75	24.14	23.76	17.10	30.06
3.3	1.00	62.48	61.18	48.54	62.11
4.6	0.38	27.39	19.60	25.89	22.65
4.6	0.63	16.20	15.93	11.91*	22.55
4.6	0.88	57.78	56.83	44.90	62.37
6.3	0.25	68.47	39.17	63.12	53.57
6.3	0.50	13.65*	10.77*	13.33	13.59*
6.3	0.75	47.54	46.83	36.78	56.03
6.3	1.00	94.88	92.29	76.87	87.45
8.5	0.38	29.04	19.09	27.75	25.63
8.5	0.63	27.85	27.16	21.05	35.08
8.5	0.88	86.27	84.08	69.59	86.41
11.3	0.25	65.48	41.32	59.57	47.46
11.3	0.50	16.95	12.83	16.17	22.81
11.3	0.75	71.48	69.62	56.71	76.84
11.3	1.00	123.94	117.72	102.78	127.06
15.1	0.38	20.55	15.09	20.29	18.97
15.1	0.63	40.15	39.63	31.91	49.04
15.1	0.88	112.99	107.67	93.08	102.16
20.0	0.25	58.38	38.74	53.10	43.78
20.0	0.50	19.70	15.66	18.49	27.97
20.0	0.75	88.34	84.90	71.31	85.81
20.0	1.00	156.74	145.39	133.24	123.72
26.4	0.38	33.41	19.14	31.85	27.14
26.4	0.63	51.61	50.59	40.34	64.53
26.4	0.88	142.32	130.19	119.50	118.80
34.7	0.25	50.46	37.19	45.97	43.82
34.7	0.50	19.61	18.20	17.41	30.38
34.7	0.75	108.45	102.38	89.26	99.95
34.7	1.00	170.39	157.28	145.63	136.35
45.7	0.38	26.45	17.04	25.72	24.98
45.7	0.63	63.29	60.97	49.86	65.04
45.7	0.88	152.24	140.22	128.85	125.50
60.0	0.25	48.40	36.51	44.21	40.11
60.0	0.50	22.13	21.02	19.36	33.01
60.0	0.75	118.41	112.63	98.08	106.80
60.0	1.00	188.28	173.84	162.76	166.80
